# Short stature and language development in the United Kingdom: a longitudinal analysis of children from the Millennium Cohort Study

**DOI:** 10.1186/s12916-022-02680-y

**Published:** 2022-12-05

**Authors:** Joseph Freer, Joanna Orr, Joan K. Morris, Robert Walton, Leo Dunkel, Helen L. Storr, Andrew J. Prendergast

**Affiliations:** 1 Queen Mary University of London, London, UK; 2 St George’s, University of London, London, UK

**Keywords:** Short stature, Stunting, Linear growth, Height, Cognition, Cognitive development, Child development

## Abstract

**Background:**

In low- and middle-income countries, poverty and impaired growth prevent children from meeting their cognitive developmental potential. There are few studies investigating these relationships in high-income settings.

**Methods:**

Participants were 12,536 children born between 2000 and 2002 in the UK and participating in the Millennium Cohort Study (MCS). Short stature was defined as having a height-for-age 2 or more standard deviations below the median (≤ − 2 SDS) at age 3 years. Standardized British Abilities Scales II (BAS II) language measures, used to assess language development at ages 3, 5, 7 and 11 years, were the main outcome assessed.

**Results:**

Children with short stature at age 3 years (4.1%) had language development scores that were consistently lower from ages 3 to 11 years (− 0.26 standard deviations (SD) (95% CI − 0.37, − 0.15)). This effect was attenuated but remained significant after adjustment for covariates. Trajectory analysis produced four distinct patterns of language development scores (low-declining, low-improving, average and high). Multinomial logistic regression models showed that children with short stature had a higher risk of being in the low-declining group, relative to the average group (relative risk ratio (RRR) = 2.11 (95% CI 1.51, 2.95)). They were also less likely to be in the high-scoring group (RRR = 0.65 (0.52, 0.82)). Children with short stature at age 3 years who had ‘caught up’ by age 5 years (height-for-age ≥ 2 SDS) did not have significantly different scores from children with persistent short stature, but had a higher probability of being in the high-performing group than children without catch-up growth (RRR = 1.84 (1.11, 3.07)).

**Conclusions:**

Short stature at age 3 years was associated with lower language development scores at ages 3 to 11 years in UK children. These associations remained significant after adjustment for socioeconomic, child and parental factors.

**Supplementary Information:**

The online version contains supplementary material available at 10.1186/s12916-022-02680-y.

## Background

Globally, children living in poverty experience impaired linear growth. Impaired growth that results in a height-for-age more than 2 standard deviations below the World Health Organization Child Growth Standards median, termed short stature or stunting, is evident in almost a quarter of children globally [1]. Although stunting is most prevalent in low- and middle-income countries (LMICs), there is increasing recognition that linear growth failure is also a marker of socioeconomic disadvantage in high-income countries (HICs) such as the United Kingdom (UK) [2–4].

In LMICs, it has been estimated that poverty and stunting prevent over 200 million children from meeting their potential for cognitive, motor and social-emotional development [5]. Data from the Young Lives study in Ethiopia, India, Peru and Vietnam have shown that linear growth from the age of 6 months is positively associated with cognition from childhood to adolescence [6–10]. A recent meta-analysis of over 9000 participants from six prospective cohorts in Brazil, Guatemala, India, the Philippines and South Africa showed statistically significant associations between linear growth in the first 1000 days (from conception to a child’s second birthday) and both schooling attainment and intelligence quotient (IQ) in adulthood [11]. This meta-analysis reinforces similar findings over the last 15 years from several other LMICs [12–22].

Given the global public health focus on countries with the highest burden of stunting, little is known about the long-term impact of poor early-life linear growth in HICs such as the UK. Longitudinal positive associations of growth and cognition have been described in HICs, but most data come from historical British birth cohorts of children born in the 1940s, which limits the applicability of these studies to current policy and clinical practice [23, 24]. Here, we use longitudinal data from the UK Millennium Cohort Study (MCS), a cohort of children born in the UK in 2000–2002. We model verbal and language development from age 3 to age 11 to assess whether short stature is associated with impaired language development in a contemporary high-income setting.

## Methods

### Sample

The MCS is an ongoing cohort study of children born between 2000 and 2002 in the UK [25–31]. The study collected data on 18,294 singleton children at the first timepoint (2000–2002). A total of 15,406 children had height and development data at two or more timepoints and were therefore eligible for analysis. A sample flowchart is presented in Additional file 1: Fig. 1. Baseline data were collected at 9 months of age (or 3 years for children not present at timepoint 1) and children were followed longitudinally with repeat data collection at ages 3, 5, 7, 11, 14 and 17 years. A stratified sampling design was used to select areas of residence (electoral wards) from which all parents of children born within the date range were invited to participate. The sampling strategy oversampled children born in ethnically diverse and economically disadvantaged areas, to account for higher attrition in these families [32]. Each data collection timepoint included an interview with the main parent (who is self-identified and is usually the mother), the parent’s resident partner if applicable and the child (starting from age 7) [32]. Child height was measured at each visit to the nearest 0.1 cm using the Leicester Measure Stadiometer (Seca Ltd, Birmingham, UK). From age 3 years, trained fieldworkers assessed verbal, language and communication abilities; numeracy; problem solving; spatial awareness; and overall school readiness using a range of validated assessments at each timepoint. The current study used data from children at ages 3, 5, 7 and 11 years, with the timepoint at 3 years old as the baseline, as well as supplemental analyses of children aged 14 and 17 years.

**Fig. 1:**
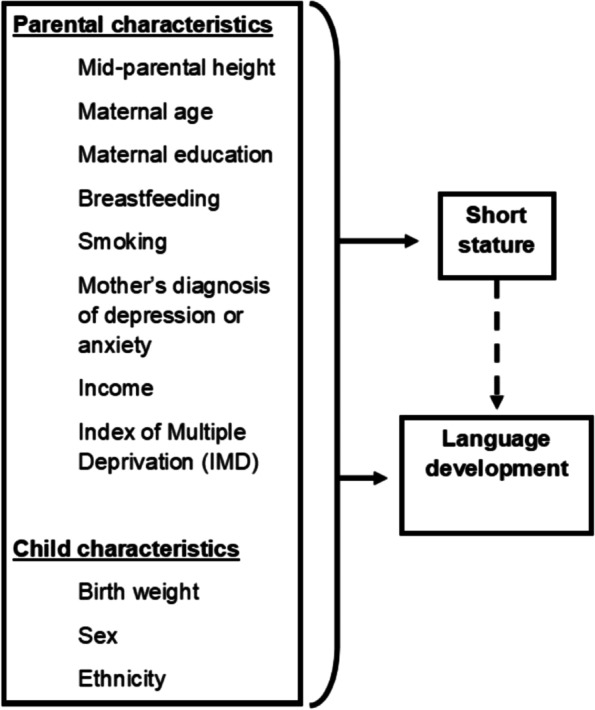
Conceptual model of the relationship between short stature and language development

Data from the MCS are accessible on request from the UK Data Service. Ethical approval for the MCS was granted by the NHS Multi-Centre Research Ethics Committee (MREC). Integrated Research Application System (IRAS) approval for the current analysis was not required.

### Measures

We used data from the British Abilities Scales II (BAS II), a battery of validated measures of verbal and language development at ages 3, 5, 7 and 11 years [33], which have been used in similar settings in the past [34]. The four timepoints available for these measures allowed us to model language development over time. These included (i) naming vocabulary (age 3 and 5 years), which assesses the child’s ability to correctly name pictures from a book; (ii) word reading (age 7 years), in which the child is asked to read aloud from a list of increasingly difficult words; and (iii) verbal similarities (age 11 years), which assesses the child’s ability to understand and describe similarities between concepts. BAS II scores are described within the MCS as age-standardised ability scores. To compare across timepoints, we further standardised these scores to have a mean of 0 and standard deviation of 1. This approach has been used previously with these data [34].

The exposure variable was short stature at age 3 years, defined as a height-for-age more than 2 standard deviations (SDS) below the population median, using UK-WHO age- and sex-specific references [35]. A broad set of variables previously reported to be associated with growth and cognitive development were examined using a directed acyclic graph (DAG; Additional file 1: Fig. 2). A parsimonious model which included child and parent characteristics available in the dataset was selected from the full set of factors presented in the DAG (Fig. 1) [3, 36–53].

**Fig. 2:**
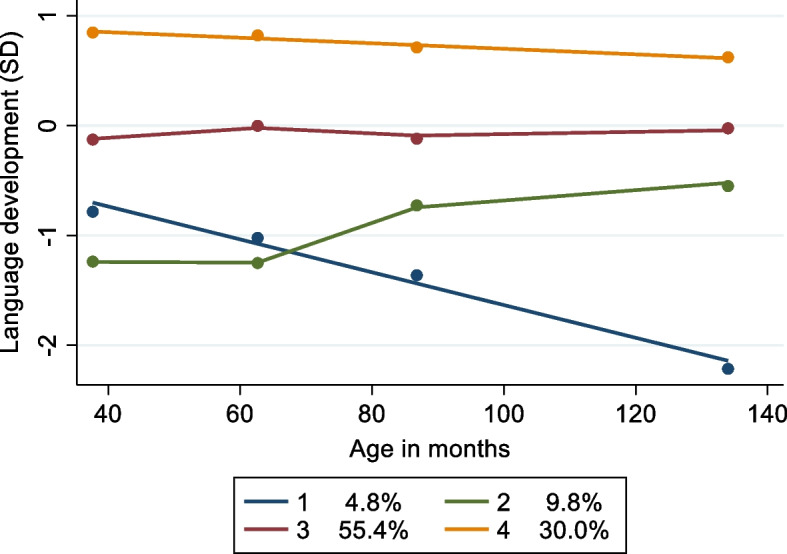
Four trajectory groups of language development between 3 and 11 years of age (*n* = 12,536). Note: dots at each timepoint reflect the within-group mean. Lines denote the within-group trajectory. The best model fit was obtained using two linear polynomic functions (groups 1 and 4) and two cubic functions (groups 2 and 3)

#### Child characteristics

Sex was collected for all children at the first interview. Birth weight in kilogrammes was collected through birth registration records. Ethnicity was recorded during the first parental interview and was coded as White ethnicity or other ethnicities, due to small numbers in non-White ethnic groups. Age was accounted for in the standardised language development and height measures. It was also included as a time variable in each model.

Children with a number of growth-affecting conditions (see Additional file 1: Table 1) were excluded from primary analyses. Growth-affecting conditions were obtained from the child’s GP through data linkage, conducted in-house at MCS, and coded using the International Classification of Diseases (ICD) code.

#### Parental characteristics

Mid-parental height was included to account for the child’s genetic height potential. Mid-parental height is calculated as the mean of mother’s and father’s standardised heights using British 1990 references [54]. Where a child had missing values for maternal or paternal height, an adjustment formula was used to impute mid-parental height based on one parental height ((available parental height *z* score × 0.4) + 0.15). This formula was derived from MCS data using the relationship between both maternal and paternal height *z* score and child’s height *z* score at age 17 years. It accounts for both the regression to the mean expected in child height compared to parental heights and the increased variance when using one parental height *z* score instead of the mean of both. We included mother’s age at birth of the child, as well as second-hand smoke exposure (“Does smoke in the same room as [child] nowadays?”) and any breastfeeding (“Did [mother] ever try to breastfeed [child]?”). Whether the mother was ever diagnosed with depression or serious anxiety was included to account for maternal mental health.

Parental socioeconomic status was assessed with a range of variables. Maternal education was included as a binary variable, with mothers who had achieved level 3 education (A levels or vocational qualification) [55] or higher categorised as having a high level of education. A levels are completed in the UK at age 18, and 34% of UK students achieved at least one A level in 1995–1996 [56]. The Index of Multiple Deprivation (IMD) is derived annually by the Office for National Statistics as a measure of small-area relative deprivation for England, Wales, Scotland and Northern Ireland [57]. Census data on seven measures (including employment, access to healthcare and environment) are compiled to create the index. In the MCS, the child’s postcode was used to provide IMD deciles, with children in the first decile being the most deprived and those in the 10th decile being the least deprived. The IMD was treated as continuous in these analyses. Finally, we included an imputed measure of income quintile available in the MCS dataset. Missing income values were estimated using interval regression and socioeconomic predictor variables [58].

### Analysis

Differences in sample characteristics by short stature were initially examined using chi-squared tests, *t* tests and ordinary least squares regression. We used two types of longitudinal growth models to assess the relationship between short stature at age 3 years and ongoing language development. Both methods used all available data, reducing the potential bias from attrition. For the current analyses, which modelled language development over time, we include participants with language development observations at two or more timepoints to adequately represent change over time.

We first assessed average language development scores over time by fitting mixed effects growth curve models using the *mixed* command in Stata 17 (StataCorp. 2021). Mixed effects models account for the highly correlated nature of repeated measures within individuals by fitting both random and fixed effects. Short stature, timepoint and covariates were included as fixed effects. Random intercept and slope effects were included by including individuals and time in the random part of the model. Time-squared and time-cubed effects were also tested as fixed effects to identify potential non-linearity in the data. Interaction terms between timepoint and short stature were included to assess whether the association with short stature changed over time. Model fit was assessed using likelihood ratio tests and assessing *p* values for individual effects. Short stature was then included in the best fitting model alone and adjusted for covariates.

We then performed group-based trajectory analysis using the *traj* user-written command in Stata [59]. Trajectory analysis uses maximum likelihood estimation to group individuals based on the similarity of their trajectories over time on the variable of interest. We used age in months as the time variable and first modelled the full sample with language development available at two or more timepoints. We used censored normal models to test the fit for 2, 3, 4 and 5 trajectory groups. We also tested the inclusion of linear, quadratic and cubic time effects. Model fit was evaluated using the Bayesian information criterion (BIC), as well as high posterior probabilities (above 0.7) and model interpretability. We then included short stature and covariates as risk in these models, as well as analysing the probability of being in each trajectory group through multinomial regression models with relative risk ratios (RRR).

Finally, we tested whether catch-up growth at age 5 (defined as having short stature at age 3 but not at age 5) was associated with improved verbal and language scores. We generated a categorical variable which included children who had never had short stature (height SDS >  − 2 at ages 3 and 5), those who had short stature at age 3 but had caught up by age 5 (height SDS ≤  − 2 at age 3, >  − 2 at age 5) and those who had persistent short stature (height SDS ≤  − 2 at ages 3 and 5). We included this variable in mixed effects models and trajectory models for language development from ages 5 to 11 years.

### Missing data

We conducted complete case analyses; children missing height or covariate data at baseline or with less than two BAS II measurements were excluded. We checked for potential bias through an examination of the characteristics of included and excluded data. We conducted sensitivity analyses using multiple imputation (described in detail in Additional file 1: Text 1).

### Sensitivity analyses

We tested a more stringent definition of short stature (≤ − 2.67 SDS), which is recommended in the UK to trigger referral for investigation into medical causes of short stature [60]. We also examined the association of height at age 3 with verbal and language development by fitting mixed effects models using a categorical variable to represent height SDS, grouping children with height-for-age of ≤  − 2 SDS, − 2 to − 1 SDS, − 1 to 0 SDS, 0 to 1 SDS, 1 to 2 SDS and > 2 SDS. A further sensitivity analysis used a later measure of language development at age 14 years in mixed effects models to assess whether the effects observed persisted into adolescence. The measure included at age 14 was the 1970 British Cohort Study Word Activity, which asks children to match a list of words to their correct synonyms. This measure was standardised in the same way as previous analyses. We did not use this measure in the main analyses as it is not part of the BAS II battery of tests. We further tested whether our results were sensitive to medical causes of short stature by excluding children who were identified as having any growth-affecting health condition (listed in Additional file 1: Table 1).

### Supplemental analyses

We also conducted a supplemental analysis using a measure of mathematical ability at age 17 to assess whether associations could be observed into later adolescence. The measure included at age 17 was the number analogy activity which assesses basic arithmetic and reasoning with numbers and is scored out of ten. We used linear regression with standardised beta coefficients to model number analogy scores and their association with short stature at age 3 years. We again adjusted for all selected covariates.

## Results

A total of 15,406 children (14,813 of 18,294 enrolled at timepoint 1 (81%), and 14,171 of 15,377 enrolled at timepoint 2 (92%)) had language development scores at two or more timepoints starting from the second wave of data collection at age 3 years; of these, 12,536 (81%) had baseline data for all covariates. Of the children included, 8799 had data for all four timepoints, with 3.9% of these children having short stature; 2474 children had data at three timepoints, with 4.9% of these children having short stature; and 1263 children had data at only two timepoints, with 6.6% of these children having short stature. A comparison of the included and excluded samples is presented in Additional file 1: Table 2, showing that there were some differences including lower BAS II scores and higher rates of short stature in the excluded sample.

A total of 514 (4.1%) children in the final sample had short stature (height SDS ≤  − 2). Sample characteristics by baseline short stature status are given in Table 1. Children with short stature had lower birth weight and younger mothers, were in a lower income quintile, lived in lower IMD areas, were less likely to have mothers with a high level of education and to have been breastfed and were more likely to be exposed to second-hand smoke.

**Table 1:** Baseline characteristics at age 3 years

Characteristic % or mean (SD)	Total	Children with short stature (height SDS ≤ − 2) (*N* = 514)	Children without short stature (height SDS > − 2) (*N* = 12,022)	*p* value*
Height, *mean (SD)*				
cm	95.5 (4.2)	87.0 (2.3)	95.9 (3.8)	< 0.001
SDS	− 0.29 (1.0)	− 2.50 (0.5)	− 0.20 (0.9)	< 0.001
Age (months), *%*	37.6 (2.4)	37.6 (2.6)	37.6 (2.4)	0.914
Sex (girls), *%*	49.4	51.0	49.3	0.462
Ethnicity (White), *%*	85.3	87.2	85.3	0.231
Birth weight (kg), *mean (SD)*	3.4 (0.6)	3.0 (0.7)	3.4 (0.6)	< 0.001
Mid-parental height SDS, *mean (SD)*	0.0 (0.8)	− 0.6 (0.8)	0.0 (0.8)	< 0.001
Mother’s age at birth of child, *mean (SD)*	29.6 (5.8)	28.9 (5.9)	29.6 (5.8)	0.012
Mother’s diagnosis of anxiety or depression, *%*	24.3	27.6	24.2	0.075
High maternal education, *%*	50.0	42.8	50.3	0.001
Second-hand smoke exposure, *%*	12.8	17.9	12.6	< 0.001
Breastfeeding, *%*	69.9	63.2	70.2	0.001
Income quintile, *%*				< 0.001
Lowest	21.0	30.2	20.6	
Second	21.6	26.7	21.4	
Third	19.7	17.7	19.7	
Fourth	19.5	12.7	19.8	
Highest	18.2	12.8	18.5	
IMD, *mean (SD)*	4.8 (2.9)	4.3 (2.8)	4.9 (2.9)	< 0.001

*IMD* Index of Multiple Deprivation decile, where 1 = most deprived and 10 = least deprived

^*^p values are for two-tailed *t* tests for continuous variables or chi-squared tests for categorical variables

### Growth curve models

Growth curve models were fitted using the full sample (*n* = 15,406, Additional file 1: Table 3) and final sample (*n* = 12,536, Additional file 1: Table 4); these were largely consistent. The best fitting growth curve model included a linear fixed time effect (timepoint). Once included in the models, short stature was negatively associated with language development (Table 2). Children with short stature at age 3 years had language scores from ages 3 to 11 that were on average − 0.26 (95% CI − 0.37, − 0.15) standard deviations below those who did not have short stature. We also included an interaction term between short stature and timepoint, which showed no significant effect, suggesting that scores did not decline or improve over time for children with short stature compared to those without short stature.

**Table 2:** Verbal and language ability random slopes and random short stature mixed effects model including short stature and timepoint interaction effect (*n* = 12,536)

		Wald chi(3) = 56.96 Prob > chi^2^ < 0.001
***Fixed effects***	Coef	95% CI
Timepoint	− 0.009*	− 0.017, − 0.002
Short stature	− 0.261***	− 0.373, − 0.150
Short stature # timepoint	0.003	− 0.035, 0.041
Constant	0.063***	0.041, 0.086
***Random effects***		
Variance (timepoint)	0.039	0.035, 0.044
Variance (constant)	0.679	0.637, 0.742
Covariance (timepoint, constant)	− 0.111	− 0.124, − 0.097
Variance (residual)	0.536	0.524, 0.547

“*” denotes coefficient is significant at *p* < 0.05 level

“**” denotes coefficient is significant at *p* < 0.01 level

“***” denotes coefficient is significant at *p* < 0.001 level

We fitted three adjusted models: the first adjusting only for sex, the second adjusting for sex and birth weight, and finally a fully adjusted model which included sex, birth weight, ethnicity, mid-parental height *z* score, mother’s age at birth of child, mother’s level of education, exposure to second-hand smoke, any breastfeeding, mother’s diagnosis of depression or anxiety, income quintile and IMD. These models showed that, whilst the effect of short stature was reduced when adjusting for covariates, short stature remained a significant predictor of poorer language development (Table 3).

**Table 3:** Verbal and language ability random slopes mixed effects models (*n* = 12,536)

	Model 1		Model 2		Model 3	
	Wald chi(4) = 98.79 Prob > chi^2^ < 0.001		Wald chi(5) = 277.67 Prob > chi^2^ < 0.001		Wald chi(17) = 3804.95 Prob > chi^2^ < 0.001	
***Fixed effects***	Coef	95% CI	95% CI	95% CI	Coef	95% CI
Timepoint	− 0.010*	− 0.017, − 0.002	− 0.010*	− 0.017, − 0.002	− 0.012**	− 0.019, − 0.004
**Short stature**	− 0.262***	** − 0.374, − 0.151**	− 0.199***	** − 0.310, − 0.088**	− 0.139**	** − 0.243, − 0.035**
Constant	− 0.061**	− 0.105, − 0.017	− 0.628	− 0.722, − 0.535	− 1.159***	− 1.266, − 1.053
***Random effects***						
Variance (timepoint)	0.039	0.035, 0.044	0.039	0.035, 0.044	0.040	0.035, 0.045
Variance (constant)	0.669	0.627, 0.714	0.651	0.609, 0.695	0.455	0.417, 0.496
Covariance (timepoint, constant)	− 0.109	− 0.122, − 0.096	− 0.107	− 0.120, − 0.094	− 0.090	− 0.103, − 0.078
Variance (residual)	0.536	0.524, 0.547	0.536	0.524, 0.547	0.535	0.524, 0.547

Verbal and language ability random slopes mixed effects model including an interaction between short stature and timepoint (not significant and not shown). Model 1 is adjusted for sex, model 2 adjusted for sex and birth weight (kg) and model 3 for sex, birth weight (kg), ethnicity, mother’s level of education, exposure to second-hand smoke, any breastfeeding, mother’s age at birth of child, mother’s diagnosis of depression or anxiety, mid-parental height SDS, income quintile and IMD. “*” denotes coefficient is significant at *p* < 0.05 level. “**” denotes coefficient is significant at *p* < 0.01 level. “***” denotes coefficient is significant at *p* < 0.001 level

### Trajectory models

The same total sample with observations at 2 or more timepoints was used to fit an initial trajectory model (*n* = 15,406). A model with four trajectory classes and including linear and cubic coefficients had the best model fit. Model fit and characteristics are presented in Additional file 1: Fig. 3 and Additional file 1: Tables 5 and 6. We then fitted the same model using the final sample (*n* = 12,536). These models were very similar; therefore, we present the final sample model here.

The four trajectory classes identified are shown in Fig. 2. The first trajectory class had the smallest membership (4.8%) and identified children with low-declining scores. The second group also had a small membership (9.8%) and low but improving scores. The third group included children with high scores (30.0%). Finally, most children had scores which were consistently around the mean (average scores) (55.4%). Variability within each trajectory class is illustrated in random sampled spaghetti plots (Additional file 1: Fig. 4). Descriptive characteristics of the children in each of these groups are given in Table 4. There was a higher percentage of children with short stature in the low scores trajectories, and a lower percentage in the high-score trajectory.

**Table 4:** Sample characteristics at baseline by trajectory group membership (*n* = 12,536)

	Low declining (498)	Low improving (979)	Average (7573)	High (3486)	Total	*p* value
Short stature age 3 years %	8.4	5.8	4.2	2.8	4.1	< 0.001
Sex (girls) %	44.2	40.6	49.3	52.8	49.4	< 0.001
Ethnicity (White) %	80.9	50.5	86.4	93.4	85.3	< 0.001
Birth weight in kg (SD)	3.3 (0.7)	3.2 (0.6)	3.4 (0.6)	3.4 (0.5)	3.4 (0.6)	< 0.001
Mid-parental height (SD)	− 0.1 (0.8)	− 0.2 (0.8)	0.0 (0.8)	0.1 (0.8)	0.0 (0.8)	< 0.001
Mother’s age, years (SD)	27.9 (6.2)	27.9 (6.0)	29.2 (5.9)	31.1 (5.2)	29.6 (5.8)	< 0.001
Mother has A level or higher %	22.9	25.7	46.3	68.7	50.0	< 0.001
Second-hand smoke exposure %	27.9	19.1	13.9	6.6	12.8	< 0.001
Breastfeeding %	55.0	65.8	66.6	80.5	69.9	< 0.001
Mother’s diagnosis of depression or anxiety	29.7	22.0	25.9	20.8	24.3	< 0.001
Income quintiles %						
Lowest	40.4	45.5	22.3	8.5	21.0	< 0.001
Second	30.5	32.9	23.7	12.7	21.6	
Third	15.3	11.0	21.2	19.4	19.7	
Fourth	9.6	6.5	18.3	27.4	19.5	
Highest	4.2	4.1	14.6	32.0	18.2	
IMD average (SD)	3.5 (2.6)	2.9 (2.2)	4.7 (2.9)	5.9 (2.8)	4.8 (2.9)	< 0.001

*p* values were determined using chi-square tests for categorical variables and ordinary least squares regression for continuous variables. There is a slight discrepancy between the trajectory percentages estimated by the model and the percentage of children in each trajectory at baseline. The model estimates the percentage of children in each trajectory based on all available data at multiple timepoints. These do not match the trajectory membership for all children with observations at baseline exactly. Due to rounding error, percentages do not always add up to 100.0

*IMD* Index of Multiple Deprivation decile, where 1 = most deprived and 10 = least deprived

Covariates were added by adjusting for risk within the trajectory class model, as well as using multinomial regression models to model the probability of being in each trajectory class. We present multinomial regression models here and adjusted risk trajectory models in Additional file 1: Table 7. Both these approaches yielded largely consistent results. The first model, adjusted for sex, showed that short stature was associated with a higher risk of being in the low-performing classes and a lower risk of being in the high-performing class. These effects remained after adjustment, although the association between short stature and a higher probability of being in the low-improving class was weak and did not persist after full adjustment for covariates (Table 5).

**Table 5:** Multinomial logistic regression model of trajectory classes by short stature and covariates (*n* = 12,536)

Trajectory class	Model 1		Model 2		Model 3	
	RRR	95% CI	RRR	95% CI	RRR	95% CI
**Average (ref)**	-	-	-	-	-	-
**Low declining**						
Short stature	2.11***	1.51, 2.95	1.88***	1.34, 2.65	1.70**	1.19, 2.42
**Low improving**						
Short stature	1.42*	1.06, 1.90	1.15	0.86, 1.55	1.23	0.90, 1.70
**High performing**						
Short stature	0.65***	0.52, 0.82	0.72**	0.57, 0.91	0.81	0.63, 1.04

Model 1 is adjusted for sex, model 2 adjusted for sex and birth weight (kg) and model 3 for sex, birth weight (kg), ethnicity, mother’s highest qualification, second-hand smoke exposure, any breastfeeding, mother’s age at birth of child, mother’s diagnosis of depression or anxiety, mid-parental height, income quintile and IMD. *RRR* relative risk ratio. RRR and 95% CI in bold are significant at the *p* < 0.05 level. “*” denotes coefficient is significant at *p* < 0.05 level. “**” denotes coefficient is significant at *p* < 0.01 level. “***” denotes coefficient is significant at *p* < 0.001 level

### Catch-up growth

Only 44.4% of children with height and full covariate data at ages 3 and 5 years who had short stature at age 3 had persistent short stature (1.8% of the whole cohort), whilst 55.6% had short stature at age 3 but not age 5 (2.2% of the whole cohort). A mixed effects model showed that catch-up growth at age 5 years was associated with some improvement in language development scores compared to no catch-up (Additional file 1: Table 8), although this effect did not reach significance (Table 6). Children with persistent short stature had lower verbal and language scores compared to children with no short stature and those who had caught up.

**Table 6:** Catch-up growth by age 5 years and language scores in children between 3 and 11 years of age (*n* = 11,860)

	Model 1		Model 2		Model 3	
	Wald chi(6) = 125.36 Prob > chi^2^ < 0.001		Wald chi(7) = 308.78 Prob > chi^2^ < 0.001		Wald chi(19) = 3718.73 Prob > chi^2^ < 0.001	
***Fixed effects***	Coef	95% CI	Coef	95% CI	Coef	95% CI
Timepoint	− 0.022	− 0.077, 0.033	− 0.021	− 0.076, 0.034	− 0.026	− 0.081, 0.029
**Catch-up growth (ref: no catch-up growth)**						
**Catch-up by 5 years**	**0.221**	** − 0.003, 0.445**	**0.180**	** − 0.043, 0.403**	**0.121**	** − 0.088, 0.330**
**No short stature**	0.372***	**0.201, 0.542**	0.286**	**0.117, 0.456**	0.187*	**0.028, 0.346**
Constant	− 0.404	− 0.577, − 0.231	− 0.877***	− 1.063, − 0.692	− 1.309***	− 1.495, − 1.123
***Random effects***						
Variance (timepoint)	0.042	0.038, 0.047	0.042	0.038, 0.047	0.042	0.038, 0.047
Variance (constant)	0.679	0.638, 0.722	0.661	0.620, 0.704	0.472	0.436, 0.511
Covariance (timepoint, constant)	− 0.115	− 0.128, − 0.103	− 0.113	− 0.126, − 0.101	− 0.097	− 0.110, − 0.085
Variance (residual)	0.520	0.510, 0.530	0.520	0.510, 0.530	0.520	0.510, 0.530

Verbal and language ability random slopes mixed effects model including an interaction between timepoint and catch-up growth, adjusted for sex (M1), for sex and birth weight (kg) (M2) and all covariates (M3). Interactions were not significant and are not shown. Catch-up growth: reference is no catch-up growth (≤ − 2 SDS at age 3 and 5 years). Catch-up by 5 years indicates short stature (≤ − 2 SDS) at age 3 years but not at age 5 years. No short stature indicates the child had height >  − 2 SDS at age 3 years

We also analysed catch-up growth using language development trajectory models. There were no significant differences in the probability of being in the low-score groups between children with and without catch-up growth. However, children with catch-up growth were significantly more likely than children without catch-up growth to be in the high-score group, although this association was attenuated after adjustment for all covariates (fully adjusted RRR = 1.65 [0.97, 2.80]) (Additional file 1: Table 9).

### Sensitivity analyses

The results of sensitivity analyses were consistent with our main analyses. Analyses by height SDS cut-offs showed that the effect of short stature on verbal and language development was fully driven by children with height SDS ≤  − 2 (Additional file 1: Table 10). A visual inspection of height SDS and standardised language development showed that the relationship between both was largely driven by an increase in language scores with height up until around height SDS − 1.5, after which the association plateaued (Additional file 1: Fig. 5). There were 138 children with very short stature (height SDS ≤  − 2.67) in the sample (1.0%) and these had lower verbal and language development scores than taller children. These effects were larger than those for short stature (height SDS ≤  − 2) (a change of − 0.43 SD compared to − 0.26 SD) (Additional file 1: Table 11). The inclusion of a further timepoint at age 14 years produced results very consistent with the 3- to 11-year models (Additional file 1: Tables 12 and 13). Excluding the 1554 (12.4% of the final sample) children with growth-affecting health conditions did not change the inference of our findings (Additional file 1: Tables 14 and 15).

Multiple imputation models produced estimates which were consistent with our final results (Additional file 1: Tables 16, 17 and 18).

### Supplemental analyses

We finally tested whether a measure of maths ability at age 17 years was associated with short stature at age 3 years (Additional file 1: Table 19). A total of 7686 children had data on all covariates at baseline as well as the Numbers Activity score at age 17. Of these, 294 (3.8%) had short stature at age 3. Regression models showed an association between short stature at age 3 and lower maths scores at age 17. Having short stature at age 3 was associated with a Numbers Activity score at age 17 that was 5% lower on average than those of children who had height SDS >  − 2. These associations were attenuated but remained significant after adjustment.

## Discussion

In this sample of 12,536 UK children participating in the Millennium Cohort Study, there were associations between short stature (also termed stunting) and poorer performance in language testing from ages 3 to 11 years. Children with short stature had language scores around a quarter of a standard deviation below those with normal height. Whilst the association of short stature attenuated after adjustment for maternal, child and deprivation factors, it remained a significant predictor of poorer language development. In trajectory modelling describing four classes of performance, short stature was associated with a higher risk of being in the two low-performing classes and a lower risk of being in the high-performing class. After adjustment, children with short stature remained significantly more likely to be in the lowest-performing group and less likely to be in the high-score group. We did not find any interactions between time and short stature, which suggests children with short stature did not improve or worsen compared to their peers. However, we want to exercise caution in interpreting this result due to the high power needed for interaction effects. Children with short stature at age 3 years who had ‘caught up’ in height by age 5 years had better language test scores than children who still had short stature at 5 years of age, but there was still a decrement in language attainment compared to children who never had short stature. Finally, we found a relationship between short stature and language development at age 14 years, and mathematical ability at age 17 years, highlighting the long-term associations between early-life short stature and school performance.

In contrast to LMICs, few recent population-level studies in HICs have investigated the relationship between linear growth and cognition, and the existing evidence is conflicting. A cross-sectional relationship between height and cognition/intelligence/academic test scores has been identified in several studies including school-age children in HICs over the last century [61–64] and evidence from the US Fragile Families study has provided evidence from a HIC that the association between height and cognition is appreciable prior to school-age [65]. Other studies in HICs have found no association between height and cognition, although these studies had small sample sizes (*n* = 91 and *n* = 221) and both recruited from single sites [66, 67]. A 2004 review of the evidence of associations between short stature and cognition highlighted the overall low quality of the available evidence including common limitations of small sample sizes and selection bias, limiting the external validity of previous studies [68],

We found that children with height SDS ≤  − 1 and height SDS ≤  − 2 both had lower language scores than children with height SDS between 0 and 1; however, after adjustment, only those with height SDS ≤  − 2 had significantly lower scores. This frames the importance of different types of growth in this twenty-first-century cohort in a high-income setting: the association appears to be driven by the ‘tail’ of children with short stature rather than height per se. This is consistent with other large studies in the USA and UK. Data from the US National Health Examination Survey in the 1960s found the association between height and cognitive test scores was most pronounced for very short children; those with heights less than the fifth percentile had significantly lower IQ than controls [63]. The UK Wessex Growth Study of children born in the 1980s showed similarly that children with heights below the 3rd centile had significantly lower scores on measures of IQ (102.6 versus 108.6; *p* < 0.005), reading attainment, and basic number skills than controls. However, contrary to these and our findings, other studies have identified a continuous association between height SDS and later outcomes without an obvious inflection point at − 1 or − 2 SD, suggesting variance in growth patterns in different settings which merits further investigation [18, 65, 69].

We found that short stature at age 3 years was longitudinally associated with a deficit in language scores of around a quarter of a standard deviation. Comparisons with other studies are challenging because of differences in methodology, as well as the age of participants and the cognitive tests employed. Nevertheless, results from supplemental analyses are broadly consistent with a 2015 meta-analysis of data from 29 LMICs, which found that each unit increase in height SDS for children over 2 years old was associated with 0.09 SD (95% CI 0.05–0.12) increase in cognitive scores [18] and also with data from the US Fragile Families cohort of 3-year-old children born between 1998 and 2000, which demonstrated 5–10% of a SD increase in cognitive test score at age 3 per SD increase in height [65]. Comparison with effect sizes from intervention studies highlights the strength of the association between poor linear growth and impaired language development outcomes among children in this UK cohort. Programmes in LMICs and HICs ranging from delivery of single interventions, such as cash transfers, to complex multi-pronged early child development packages have reported effect sizes of between 0.1 and around 0.4 [70, 71].

Given the vital importance of the first 1000 days to linear growth and brain development [72], the question of whether catch-up growth and development are possible after this ‘window period’ has been the subject of debate. Evidence from multiple LMICs has demonstrated that whilst the first 1000 days are critical to cognitive, motor and social-emotional development, linear growth after 2 years of age appears either not associated or significantly more weakly associated with cognitive scores and academic outcomes [16, 18, 73–77]. The potential for catch-up development among children with short stature is important because without intervention, children with delayed cognitive development are more likely to fall behind throughout subsequent education and employment [78–80]. Using mixed effects linear models adjusted for maternal, child and deprivation factors, we found that although children with and without catch-up growth both had lower language development scores than children who did not have short stature, these were not significantly different from each other. However, children who had height-for-age ≤  − 2 at age 3 years, but > 2 at age 5 years had higher language development scores and a higher probability of being in the high scores trajectory group. We chose 5 years as the age to re-assess language scores, as the potential for ‘catch-up’ growth and development is thought to be greater in early life [81]. We considered the potential for some of these children to have ‘regressed to the mean’ rather than ‘caught-up’ [82]. However, the inclusion of all children who had short stature at age 3 years but not at age 5 years is a more conservative approach to estimate these effects.

The mechanisms for the association between linear growth in early life and downstream cognitive, developmental, educational and economic outcomes are not fully established. Moreover, much of the research interrogating the intermediary pathways has taken place in LMICs, so the findings of that body of research—especially relating to severe malnutrition, sanitation, infection burden and immune dysfunction—might not be generalisable. However, a recent analysis of national data in England identified a strong linear association between short stature and deprivation, and Table 1 demonstrates a similar pattern among MCS children [83]. Together, these results suggest that the well-documented interactions between poverty and growth in historical HIC cohorts and LMICs hold true in contemporary high-income settings. In the MCS cohort, short stature at age 3 years remained a predictor for impaired language development even after adjustment for several socioeconomic variables. This could represent a direct effect, residual confounding or a combination of both. We did not directly investigate mechanisms, but children with short stature in this cohort had lower birth weight, were less likely to have mothers with a high level of education, were more likely to be exposed to second-hand smoke, were less likely to have had any breastfeeding, had younger mothers and a lower (i.e. more deprived) index of multiple deprivation. Further research is required to investigate the pathways between growth, development and child poverty in both high-income and LMIC settings.

This study had several strengths including the relevance to policy and practice of a large national cohort of children born in the twenty-first century. This is also the first of the four national British birth cohort studies to have taken anthropometric measurements from all children at the age of 3 years and we investigated longitudinal associations up to the age of 17, which represents a follow-up to an older age than has been investigated by most earlier studies. The sampling strategy also overcomes a serious shortcoming of many previous studies in high-income countries, which have often been subject to referral bias. The study also had several limitations. The earliest height measurement in the MCS data was taken at age 3, and the most recently published data was from participants at age 17. We were therefore unable to assess linear growth patterns in the first 1000 days, during which period growth is most sensitive to the environment [84]. We were also unable to investigate whether the associations persist into adulthood. We adjusted for a large number of covariates, but the non-availability of some factors in the DAG (Additional file 1: Fig. 2) could have resulted in residual confounding. Furthermore, data on birth length were not available; whilst birth weight and birth length are correlated, there is some evidence that length is more directly associated with height in later life [85]. However, in practice, birth length measures are often inaccurate [86], and the correlation between birth weight and birth length could have resulted in collinearity if they had both been included in the models. We observed differences between our excluded and included samples, suggesting that those who were excluded from the current analyses had worse verbal and language development scores and higher prevalence of short stature, as well as being more likely to experience other adverse circumstances such as exposure to second-hand smoke, no breastfeeding and higher levels of deprivation. Although these children represent a small group, it is possible that we underestimated the relationship between short stature and language development, because of the socioeconomic vulnerability of the excluded group.

## Conclusions

Whilst the observational nature of the study limits possible conclusions about any causal relationships between short stature and cognition, the strength of the longitudinal association after adjustment for socioeconomic confounders is striking. Short stature at age 3 years or younger could potentially serve as a marker of future risk of cognitive or educational problems and be used to identify children who would benefit from further assessment and early intervention. Such a marker could have several benefits over other predictive factors for impaired cognition in later life. Height is relatively simple and inexpensive to measure, interpretation is straightforward and linear growth monitoring in childhood is already embedded in public healthcare in most countries [87, 88]. A routine height measurement is also potentially less stigmatising than assessment for other markers of poverty. In contrast, many countries (including the UK) only measure children’s heights routinely at school entry, and further research is required to establish the feasibility, acceptability and utility of pre-school growth screening in high-income countries.

Since most children with short stature will not have impaired cognitive development, the challenge for a successful pre-school growth-screening programme is to sensitively identify the children who could benefit from early intervention without trading off specificity and causing harm through unnecessary investigation. Equally, it is important not to medicalise and stigmatise individual children’s ‘short but normal’ growth. Although short stature appears to be an important predictor of future cognition, it might not be sufficient to identify the children most in need. Further research is required to establish the variables which could be used in combination with linear growth to accurately identify children who would benefit from early intervention to support cognitive development.

## Supplementary Information

**Additional file 1.** Contains the study’s sampling flowchart; a Directed Acyclic Graph; details of our missing data handling and imputation strategy; and details of our supplemental analyses.

## Data Availability

Data from the MCS are accessible on request from the UK Data Service.

## Abbreviations

DAG: Directed acyclic graph
HICs: High-income countries
IMD: Index of Multiple Deprivation
IQ: Intelligence quotient
LMICs: Low- and middle-income countries
MCS: Millennium Cohort Study
