# Supplementary material for: Short stature and language development in the United Kingdom: a longitudinal analysis of children from the Millennium Cohort Study
Source: BMC Med. 2022 Dec 5;20:468. doi: 10.1186/s12916-022-02680-y (PMC9721056; doi:10.1186/s12916-022-02680-y)
Supplement: Supplementary file 1 — Additional file 1. Contains the study’s sampling flowchart; a Directed Acyclic Graph; details of our missing data handling and imputation strategy; and details of our supplemental analyses. [file 12916_2022_2680_MOESM1_ESM.docx]

Fig 1. Sample flow chart


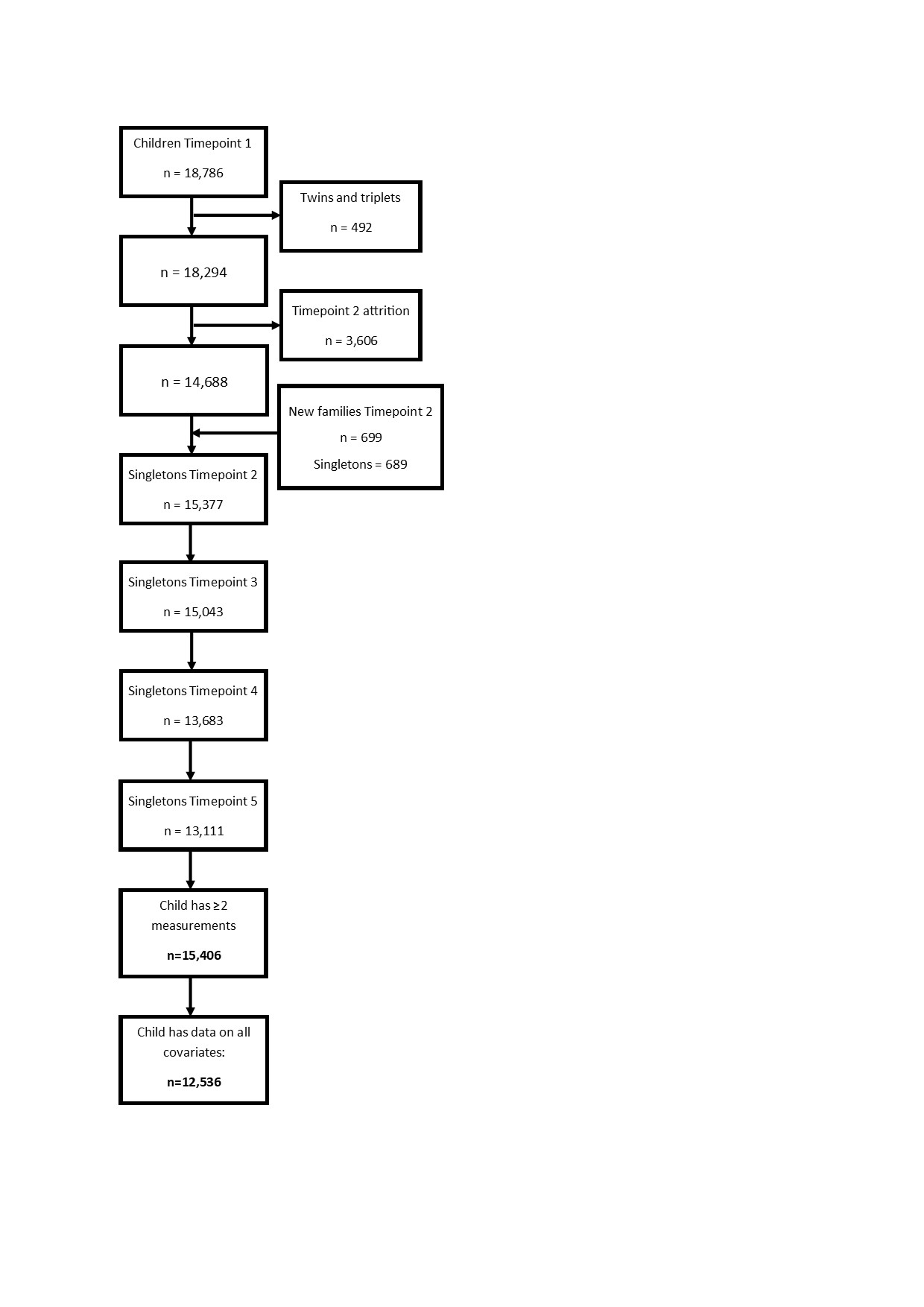


Note: The number of children with ≥2 measurements is higher than the number of children at Sweep 2. This is because children who did not participate in Sweep 2 but did participate at Sweep 1 and at least two subsequent sweeps were eligible for analysis, as baseline data was obtained at Sweep 1.

Fig 2. Directed Acyclic Graph


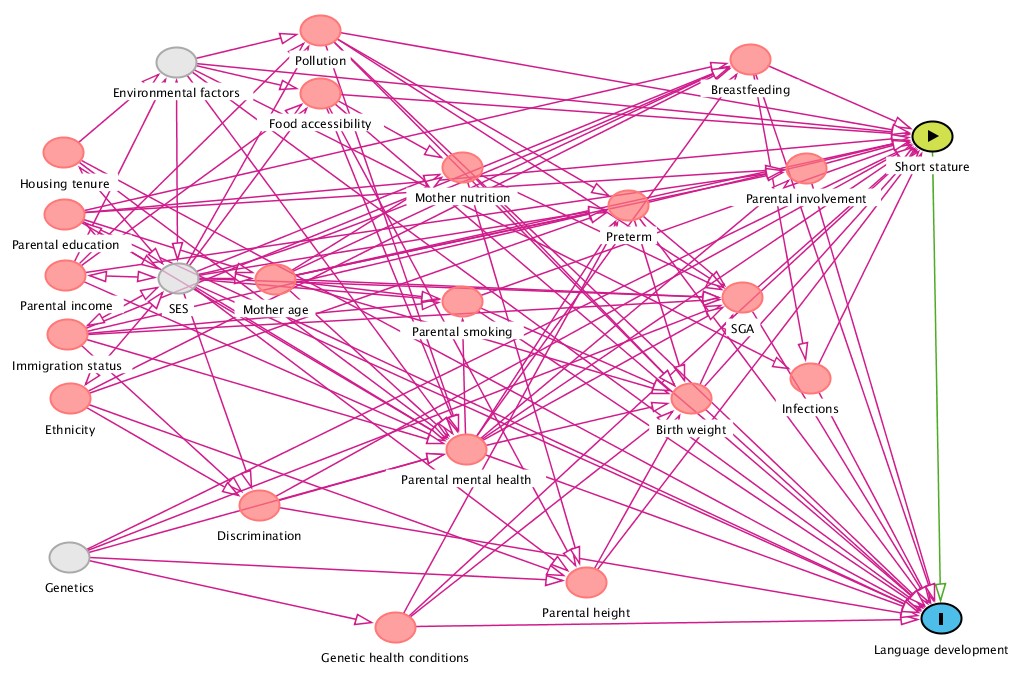


Note: Blue oval denotes outcome. Green oval denotes exposure. Grey ovals denote latent (unobserved) variables. Pink ovals denote potential adjustment variables. All potential adjustment variables are considered to be confounders. No mediators (pathways between short stature exposure and outcome language development) were considered within the model. Arrows denote direction of potential effect. Model available on dagitty.net/moSFF3R.

References in the following paragraphs refer to the reference list in the main text. Associations have been previously shown between cognitive development and birth weight, prematurity and small-for-gestational age (SGA; birth weight below the 10^th^ centile for gestational age) (36-38), childhood infections (39), breastfeeding (40, 41), parental involvement (42), parental (in particular maternal) mental health (43, 44), parental smoking (45), mother’s age(46), a range of socioeconomic status markers(47, 48), and experiences of discrimination (49).

Growth has been shown to be associated with many of the same variables in Lower and Middle Income Countries (LMIC) and in High Income Countries (HIC). Markers of Socioeconomic Status (SES) have been shown consistently to be associated with poorer growth (50-52), as has birth weight, preterm birth and SGA (53, 54). Some factors have been shown to be important in LMICs but do not have the same level of evidence in HICs, such as childhood infections and maternal nutrition (3). Other factors, such as breastfeeding and pollution, have mixed evidence in both LMIC and HICs (3, 53).

Text 1. Missing data handling

We judged that missing data in our sample were missing at random (MAR); data were more likely to be missing from children living in more deprived areas and whose mothers had lower levels of education (See Additional file 1: Table 2 for a more detailed breakdown or included and excluded samples). We therefore used household and area level SES characteristics to adjust for this potential bias in models. Furthermore, children with missing data were shorter and had lower BAS II scores. To reduce potential bias, we first fitted each model on a ‘full’ sample of children with two or more BAS II measurements and followed-up using the final sample of children with data on all covariates. We present both models. We also present a comparison of sample characteristics for included and excluded samples.

Imputation strategy

A sensitivity analysis using multiple imputation was conducted to ensure potential biases arising from missing data were not overlooked. We assumed data were missing at random, with data missingness largely predicted by socio-economic and regional variables which were observed in the data. We used multiple imputation using chained equations (MICE) in Stata. MICE takes into account potential differences in the distribution of auxiliary and predicted variables. A total of forty-five imputed datasets were added, and data were ‘long’, for compatibility with mixed effects modelling. Additional file 1: Table 16 shows predicted and auxiliary variables, the numbers missing and imputed, and the distribution of each variable.

Complete auxiliary variables were used when these were present in the models (sex, timepoint, diagnosed health conditions) and when they helped predict missingness (regions). Incomplete auxiliary variables were also used. These were selected if they were predictive of either missingness or other variables of interest. These included the Bracken School Readiness Assessment-Revised (BSRA-R) which was used to assess basic knowledge in preparation for school at age 3 (timepoint 2). This was included in the imputation model as an age-adjusted percentile. We also included weight-for-age z score at all available time points as an auxiliary variable. This weight-for-age z score was included to help predict the child’s height at age 3 years.

Table 1. Growth affecting health conditions.

| Health condition | ICD code |
| --- | --- |
| Anaemia | D50-D64 |
| Immunodeficiency | D82 |
| Hypothyroidism | E03 |
| Growth Hormone Deficiency | E23 |
| Other endocrine disorders | E34 |
| Malnutrition | E40-E46 |
| Metabolism disorders | E76-E77 |
| Cystic Fibrosis | E84 |
| Diseases of myoneural junction and muscle | G70-G73 |
| Chronic lower respiratory diseases | J40-J47 |
| Lung diseases | J60-J70 |
| Other respiratory diseases | J80-J99 |
| Noninfective enteritis and colitis | K50-K52 |
| Liver diseases | K70-K77 |
| Other diseases of the digestive system | K90-K93 |
| Arthritis | M08 |
| Kidney disease | N18 |
| Impaired renal tubular function | N25 |
| Fetus affected by maternal conditions | P00 |
| Poor fetal growth | P05-P07 |
| Congenital malformations of the nervous system | Q00-Q07 |
| Congenital malformations of the musculoskeletal system | Q65-Q79 |
| Other congenital malformations | Q80-89 |
| Chromosomal abnormalities | Q90-Q99 |

Table 2. Total eligible, included and excluded sample comparison.

|  | Total  (18,981) | Excluded | Included (unadjusted models)  (n=15,406) | Included (adjusted models) (n=12,536) |
| --- | --- | --- | --- | --- |
| BAS score age 3 mean (SD) | 0·00 (1·00)  (14,372) | -0·15 (1·01)  (934) | 0·01 (1·00)  (13,438) | 0·04 (0·98)  (12,066) |
| BAS score age 5 mean (SD) | 0·00 (1·00)  (15,042) | -0·88 (1·37)  (391) | 0·02 (0·98)  (14,651) | 0·08 (0·95)  (11,964) |
| BAS score age 7 mean (SD) | 0·00 (1·00)  (13,243) | -0·32 (0·98)  (73) | 0·00 (1·00)  (13,170) | 0·03 (0·99)  (10,754) |
| BAS score age 11 mean (SD) | 0·00 (1·00)  (12,823) | -0·48 (1·26)  (181) | 0·01 (0·99)  (12,642) | 0·03 (0·98)  (10,360) |
| Short stature (age 3) | 4·2  (14,298) | 6·6  (1,034) | 4·1  (13,264) | 4·1  (12,536) |
| Height (age 3) (cm)* | 95·6 (4·3)  (14,306) | 95·8 (4·8)  (1,035) | 95·5 (4·2)  (13,271) | 95·5 (4·2)  (12,536) |
| Height (age 3) (SDS)* | -0·29 (1·01)  (14,298) | -0·32 (1·11)  (1,034) | -0·29 (1·01)  (13,264) | -0·29 (1·01)  (12,536) |
| Sex % |  |  |  |  |
| Boys | 51·5  (18,981) | 52·9  (3,575) | 51·2  (15,406) | 50·6  (12,536) |
| Girls | 48·5  (18,981) | 47·1  (3,575) | 48·8  (15,406) | 49·4  (12,536) |
| Ethnicity (White) | 81·9  (18,862) | 77·0  (3,549) | 83·1  (15,313) | 85·3  (12,536) |
| Mother has A level or higher % | 47·0  (18,892) | 38·0  (3,535) | 48·5  (14,779) | 50·0  (12,536) |
| Exposed to second-hand smoke % | 13·6  (18,268) | 14·6  (3,470) | 13·4  (14,798) | 12·8  (12,536) |
| Breastfeeding % | 67·1  (18,240) | 58·6  (3,457) | 69·1  (14,783) | 69·9  (12,536) |
| Mother’s diagnosis of depression or anxiety | 24·6  (18,269) | 24·1  (3,472) | 24·7  (14,797) | 24·3  (12,536) |
| Income quintile % |  |  |  |  |
| Lowest | 25·2  (18,212) | 33·4  (3,443) | 23·2  (14,769) | 21·0  (12,536) |
| Second | 22·5  (18,212) | 25·6  (3,443) | 21·8  (14,769) | 21·6  (12,536) |
| Third | 18·9  (18,212) | 18·1  (3,443) | 19·1  (14,769) | 19·7  (12,536) |
| Fourth | 17·4  (18,212) | 12·3  (3,443) | 18·6  (14,769) | 19·5  (12,536) |
| Highest | 16·0  (18,212) | 10·6  (3,443) | 17·2  (14,769) | 18·2  (12,536) |
| Mother’s age at birth of child (SD) | 29·1 (6·0)  (18,243) | 28·1 (6·0)  (3,463) | 29·3 (5·9)  (14,780) | 29·6 (5·8)  (12,536) |
| Mid-parental height SDS (SD) | 1. (0·8)   (18,714) | -0·0 (0·7)  (3,501) | 1. (0·8)   (15,213) | 0·0 (0·8)  (12,536) |
| Birth weight (SD) (kg) | 3·4 (0·6)  (18,304) | 3·3 (0·6)  (3,476) | 3·4 (0·6)  (14,828) | 3·4 (0·6)  (12,536) |
| IMD average (SD) | 4·6 (2·9)  (18,294) | 4·1 (2·8)  (3,481) | 4·7 (2·9)  (14,813) | 4·8 (2·9)  (12,536) |

Note: there is a small discrepancy in the sample sizes for height and height SDS due to 6 children having extreme heights which fell outside the ranges specified for the calculation of UK-WHO references (-4.6 to 4.6 SDS).

Total sample size was all eligible singletons with data at Timepoints 1 or 2 (n=18,981).

Excluded children were those with language development measures at less than two time points.

Table 3. Verbal and language ability random intercepts mixed effects model, full sample (n=15,406).

|  |  | Wald chi(1) = 0·07  Prob > chi2 = 0·7892 |
| --- | --- | --- |
| ***Fixed effects*** | Coef. | 95% CI |
| Timepoint | -0·001 | -0·008, 0·006 |
| Constant | -0·007 | -0·028, 0·013 |
| ***Random effects*** |  |  |
| Variance (timepoint) | 0·040 | 0·036, 0·045 |
| Variance (constant) | 0·728 | 0·688, 0·771 |
| Covariance (timepoint, constant) | -0·117 | -0·129, -0·105 |
| Variance (residual) | 0·547 | 0·538, 0·557 |

Table 4. Verbal and language ability random intercepts mixed effects model, final sample (n=12,536).

|  |  | Wald chi(1) = 6·47  Prob > chi2 = 0·0110 |
| --- | --- | --- |
| ***Fixed effects*** | Coef. | 95% CI |
| Timepoint | -0·009* | -0·016, -0·002 |
| Constant | 0·053*** | 0·031, 0·075 |
| ***Random effects*** |  |  |
| Variance (timepoint) | 0·039 | 0·035, 0·044 |
| Variance (constant) | 0·682 | 0·639, 0·727 |
| Covariance (timepoint, constant) | -0·111 | -0·124, -0·097 |
| Variance (residual) | 0·536 | 0·524, 0·547 |

Figure 3. Comparison of full sample trajectory model and final sample trajectory model· Four class solution, full sample (A) (n=15,406) and final sample (B) (12,536).

| A   | B   |
| --- | --- |

Table 5. Trajectory group characteristics as measured at baseline (n=15,406).

|  | Group 1  (low decliners) | Group 2  (low improvers) | Group 3  (average) | Group 3  (high performers) | Total  (Sample n) |
| --- | --- | --- | --- | --- | --- |
| % girls in class | 42·0 | 41·0 | 48·2 | 52·8 | 48·8  (15,406) |
| % white ethnicity | 79·6 | 41·2 | 83·5 | 92·0 | 83·1  (15,313) |
| % short stature at age 3 in class | 8·4 | 5·8 | 4·1 | 2·9 | 4·1  (13,264) |
| % mother has A level or higher | 23·6 | 28·1 | 41·7 | 67·1 | 49·1  (15,357) |
| % second-hand smoke | 27·7 | 17·4 | 14·8 | 7·6 | 13·4  (14,798) |
| % breastfeeding | 53·1 | 67·3 | 65·0 | 79·8 | 69·1  (14,783) |
| % mother ever diagnosed with depression or anxiety | 31·5 | 20·3 | 26·3 | 21·5 | 24·7  (14,797) |
| Mother’s age at birth of child (SD) | 27·8 (6·1) | 27·8 (5·9) | 28·8 (6·0) | 31·0 (5·3) | 29·3 (5·9)  (14,780) |
| Mid-parental height (SDS) | -0·13 (0·8) | -0·31 (0·8) | -0·01 (0·8) | 0·13 (0·8) | 0·01 (0·8)  (15,213) |
| Birth weight (SD) (kg) | 3·2 (0·7) | 3·2 (0·6) | 3·4 (0·6) | 3·4 (0·5) | 3·4 (0·6)  (14,828) |
| Income quintile % |  |  |  |  |  |
| Lowest | 44·1 | 50·0 | 25·7 | 9·8 | 23·2  (3,429) |
| Second | 30·9 | 30·7 | 24·3 | 13·9 | 21·8  (3,222) |
| Third | 12·7 | 9·7 | 20·4 | 19·6 | 19·1  (2,826) |
| Fourth | 8·1 | 6·3 | 16·7 | 26·4 | 18·6  (2,748) |
| Highest | 4·2 | 3·3 | 13·0 | 30·3 | 17·2  (2,544) |
| IMD average (SD) | 3·4 (2·5) | 2·6 (2·1) | 4·5 (2·9) | 5·7 (2·9) | 4·7 (2·9)  (14,813) |

Note: the model estimates the percentage of in each class membership, and they are based on the use of all available data at multiple time points. These do not match the class membership for all children with observations at baseline exactly.

Table 6. Model fit comparisons.

| Sample size | **BIC** | **Entropy** | **Posterior probabilities** |
| --- | --- | --- | --- |
| 15,406 | -71739·82 | 0·67 | 0·73; 0·82 |
| 12,536 | -59447·36 | 0·66 | 0·72; 0·82 |

Fig 4. Spaghetti plots of standardized language development over four time points in each trajectory group.

| Group 1: Low declining | Group 2: Low improving |
| --- | --- |
|  |  |
| Group 3: Average | Group 4: High |
|  |  |

Table 7. Trajectory class model adjusted for short stature and covariates (n=12,536).

| Trajectory class | Model 1 | | Model 2 | | Model 3 | |
| --- | --- | --- | --- | --- | --- | --- |
|  | Estimate | P value | Estimate | P value | Estimate | P value |
| Average (ref) | - | - | - | - | - | - |
| Low declining |  |  |  |  |  |  |
| Short stature | **0·878** | **<0·001** | **0·773** | **0·002** | **0·586** | **0·001** |
| Low improving |  |  |  |  |  |  |
| Short stature | **0·498** | **0·009** | **0·226** | **0·253** | **0·719** | **0·070** |
| High performing |  |  |  |  |  |  |
| Short stature | **-0·536** | **0·004** | **-0·422** | **0·021** | **-0·396** | **0·029** |

Note: Model 1 is adjusted for sex, Model 2 adjusted for sex and birth weight (kg), and Model 3 for sex, birth weight (kg), ethnicity, mother’s highest qualification, any parental smoking, any breastfeeding, mother’s age at birth of child, mother’s depression or anxiety diagnosis, mid-parental height SDS, income and IMD.

Table 8. Trajectory class and catch-up growth (n=11,860).

|  | Class 1  (low declining) | Class 2  (low improving) | Class 3  (average) | Class 3  (high) | Total |
| --- | --- | --- | --- | --- | --- |
| % no short stature at age 3 years | 91·2 | 94.0 | 95·9 | 97·3 | 95·9 |
| % catch up | 4·1 | 3·3 | 2·3 | 1·8 | 2·3 |
| % no catch up | 4·7 | 2·8 | 1·9 | 0·8 | 1·8 |

Table 9. Multinomial logistic regression models of trajectory classes adjusted for catch-up growth and covariates (n=11,860).

| Trajectory class | Model 1 | | Model 2 | | Model 3 | |
| --- | --- | --- | --- | --- | --- | --- |
|  | RRR | 95% CI | RRR | 95% CI | RRR | 95% CI |
| Average (ref) | - | - | - | - | - | - |
| Low declining |  |  |  |  |  |  |
| Catch up | 0.71 | 0·36, 1.39 | 0.77 | 0·39, 1·50 | 0.80 | 0.41, 1.60 |
| No short stature | **0.37** | **0.23, 0.60** | **0.44** | **0·27, 0·71** | **0·51** | **0·31, 0·83** |
| Low improving |  |  |  |  |  |  |
| Catch up | 0.97 | 0·56, 1.67 | 1·11 | 0·64, 1·93 | 1.26 | 0.70, 2.26 |
| No short stature | **0.65** | **0.43, 0.98** | 0.86 | 0·57, 1·31 | 0.90 | 0.57, 1.40 |
| High |  |  |  |  |  |  |
| Catch up | **1.84** | **1.11, 3.07** | **1·73** | **1·04, 2·88** | 1.65 | 0·97, 2.80 |
| No short stature | **2.30** | **1.51, 3.48** | **2.01** | **1.32, 3·05** | **1.72** | **1.11, 2·66** |

Note: Model 1 is adjusted for sex, Model 2 adjusted for sex and birth weight (kg), and Model 3 for sex, birth weight (kg), ethnicity, mother’s highest qualification, any parental smoking, any breastfeeding, mother’s diagnosis of depression or anxiety, mother’s age at birth of child, mid-parental height, income quintile and IMD.

Table 10. SDS cut offs and language development in children ages 3-11 years (n=12,536).

|  | Model 1 | | Model 2 | | Model 3 | |
| --- | --- | --- | --- | --- | --- | --- |
|  | Wald chi(12) = 178·31  Prob > chi2 <0·001 | | Wald chi(13) = 318·82  Prob > chi2 <0·001 | | Wald chi(25) = 39846·81  Prob > chi2 <0·001 | |
| ***Fixed effects*** | Coef· | 95% CI | Coef· | 95% CI | Coef· | 95% CI |
| Timepoint | -0·005 | -0·018, 0·008 | -0·005 | -0·018, 0·008 | -·007 | -0·021, 0·006 |
| **SDS cut offs**  (0 to 1 ref) |  |  |  |  |  |  |
| <-2 | **-0·299***** | **-0·416, -0·182** | **-0·229***** | **-0·345, -0·113** | **-0·166**** | **-0·276, -0·056** |
| -2 to -1 | **-0·104**** | **-0·169, -0·039** | -0·065 | -0·129, 0·000 | -0·042 | -0·104, 0·019 |
| -1 to 0 | **-0·040** | **-0·094, 0·013** | -0·024 | -0·077, 0·029 | -0·014 | -0·064, 0·036 |
| 1 to 2 | **-0·014** | **-0·078, 0·106** | -0·004 | -0·095, 0·088 | -0·019 | -0·066, 0·104 |
| >2 | **-0·044** | **-0·240, 0·154** | -0·067 | -0·263, 0·128 | -0·021 | -0·200, 0·157 |
| Constant | -0·028 | -0·084, 0·028 | -0·555*** | -0·658, -0·452 | -1·120*** | -1·233, -1·007 |
| ***Random effects*** |  |  |  |  |  |  |
| Variance (timepoint) | 0·039 | 0·035, 0·044 | 0·039 | 0·035, 0·044 | 0·039 | 0·035, 0·045 |
| Variance (constant) | 0·668 | 0·626, 0·713 | 0·651 | 0·610, 0·696 | 0·454 | 0·416, 0·495 |
| Covariance (timepoint, constant) | -0·109 | -0·122, -0·096 | -0·107 | -0·120, -0·094 | -0·090 | -0·103, -0·078 |
| Variance (residual) | 0·536 | 0·524, 0·547 | 0·536 | 0·524, 0·547 | 0·535 | 0·524, 0·547 |

Note: Verbal and language ability random slopes mixed effects model for SDS cut offs with average as reference, including an interaction effect between timepoint and SDS cut off (not significant and not shown) and sex (M1), birth weight (M2) and all covariates (M3).

Fig. 5. Scatterplot of height SDS and standardized language development at all timepoints.

Table 11. Very short stature (SDS < -2·67) and language development at ages 3-11 years (n=12,536).

|  | Model 1 | | Model 2 | | Model 3 | |
| --- | --- | --- | --- | --- | --- | --- |
|  | Wald chi(4) = 73·64  Prob > chi2 <0·001 | | Wald chi(5) = 265·49  Prob > chi2 <0·001 | | Wald chi(17) = 3800·98  Prob > chi2 <0·001 | |
| ***Fixed effects*** | Coef· | 95% CI | Coef· | 95% CI | Coef· | 95% CI |
| Timepoint | -0·010** | -0·017, -0·002 | -0·010** | -0·017, -0·002 | -0·012** | -0·019, -0·004 |
| **Very short stature (SDS <-2·67)** | **-0·429***** | **-0·665, -0·193** | **-0·347**** | **-0·582, -0·112** | **-0·271*** | **-0·493, -0·050** |
| Constant | -0·067** | -0·111, -0·023 | -0·649*** | -0·742, -0·555 | -1·168*** | -1·275, -1·062 |
| ***Random effects*** | |  |  |  |  |  |
| Variance (timepoint) | 0·039 | 0·035, 0·044 | 0·039 | 0·035, 0·044 | 0·040 | 0·035, 0·045 |
| Variance (constant) | 0·670 | 0·628, 0·715 | 0·651 | 0·609, 0·695 | 0·455 | 0·417, 0·496 |
| Covariance (timepoint, constant) | -0·109 | -0·122, -0·096 | -0·107 | -0·120, -0·094 | -0·090 | -0·102, -0·078 |
| Variance (residual) | 0·536 | 0·524, 0·547 | 0·536 | 0·524, 0·547 | 0·535 | 0·524, 0·547 |

Note: 138 children had very short stature (1·0%). Verbal and language ability random slopes mixed effects model including an interaction between timepoint and very short stature (not significant and not shown), adjusted for sex (Model 1), for sex and birth weight (kg) (Model 2) and for all covariates (Model 3).

Table 12. Verbal and language ability random slopes mixed effects model up to age 14, by short stature, final adjustment sample (n=12,592).

|  |  | Wald chi(2) = 73·69  Prob > chi2 < 0·001 |
| --- | --- | --- |
| ***Fixed effects*** | Coef· | 95% CI |
| Timepoint | -0·013*** | -0·018, -0·007 |
| Short stature | -0·249*** | -0·315, -0·182 |
| Constant | 0·068*** | 0·048, 0·088 |
| ***Random effects*** |  |  |
| Variance (timepoint) | 0·024 | 0·021, 0·027 |
| Variance (constant) | 0·586 | 0·552, 0·621 |
| Covariance (timepoint, constant) | -0·074 | -0·082, -0·065 |
| Variance (residual) | 0·561 | 0·551, 0·571 |

Table 13. Verbal and language ability random slopes mixed effects model up to age 14 (n=12,592).

|  | Model 1· | | Model 2· | | Model 3· | |
| --- | --- | --- | --- | --- | --- | --- |
|  | Wald chi(4) = 114·13  Prob > chi2 <0·001 | | Wald chi(5) = 285·40  Prob > chi <0·001 | | Wald chi(16) = 3856·60  Prob > chi2 <0·001 | |
| ***Fixed effects*** | Coef· | 95% CI | Coef· | 95% CI | Coef· | 95% CI |
| Timepoint | -0·013*** | -0·019, -0·008 | -0·013 | -0·019 -0·008 | -0·015*** | -0·021, -0·010 |
| **Short stature** | **-0·286***** | **-0·390, -0·183** | **-0·226** | **-0·329, -0·124** | **-0·176***** | **-0·271, -0·081** |
| Constant | -0·048** | -0·090, -0·006 | -0·579 | -0·669, -0·489 | -1·129*** | -1·231, -1·027 |
| ***Random effects*** |  |  |  |  |  |  |
| Variance (timepoint) | 0·024 | 0·021, 0·027 | 0·024 | 0·021, 0·027 | 0·024 | 0·022, 0·027 |
| Variance (constant) | 0·578 | 0·544, 0·613 | 0·561 | 0·528, 0·596 | 0·387 | 0·357, 0·419 |
| Covariance (timepoint, constant) | -0·073 | -0·081, -0·064 | -0·071 | -0·079, -0·062 | -0·059 | -0·067, -0·051 |
| Variance (residual) | 0·561 | 0·551, 0·571 | 0·561 | 0·551, 0·571 | 0·561 | 0·551, 0·571 |

Verbal and language ability random slopes mixed effects model including an interaction between timepoint and short stature (not significant and not shown), adjusted for sex (Model 1), for sex and birth weight (kg) (Model 2) and for all covariates (Model 3).

Table 14. Verbal and language ability random slopes mixed effects model excluding children with growth-affecting health conditions (n=10,982).

|  |  | Wald chi(3) = 37·75  Prob > chi2 < 0·001 |
| --- | --- | --- |
| ***Fixed effects*** | Coef· | 95% CI |
| Timepoint | -0·007 | -0·015, 0·001 |
| Short stature | -0·252*** | -0·373, -0·131 |
| Constant | 0·068*** | 0·044, 0·092 |
| ***Random effects*** |  |  |
| Variance (timepoint) | 0·040 | 0·035, 0·045 |
| Variance (constant) | 0·690 | 0·645, 0·739 |
| Covariance (timepoint, constant) | -0·115 | -0·129, -0·101 |
| Variance (residual) | 0·534 | 0·522, 0·546 |

Note: There were 1,891 children in the full sample, and 1,554 in children in the final sample with health conditions which affect growth (see Additional file 1: Table 1)· An interaction effect between short stature and timepoint was included in model but was not significant and is not shown·

Table 15. Verbal and language ability random slopes mixed effects model excluding children with growth-affecting health conditions (n=10,982).

|  | Model 1· | | Model 2· | | Model 3· | |
| --- | --- | --- | --- | --- | --- | --- |
|  | Wald chi(4) = 75·96  Prob > chi2 <0·001 | | Wald chi(5) = 243·48  Prob > chi2<0·001 | | Wald chi(17) = 3371·93  Prob > chi2 <0·001 | |
| ***Fixed effects*** | Coef· | 95% CI | Coef· | 95% CI | Coef· | 95% CI |
| Timepoint | -0·007 | -0·015, 0·001 | -0·007 | -0·015, 0·001 | -0·009* | -0·017, -0·001 |
| **Short stature** | **-0·253***** | **-0·374, -0·132** | **-0·193**** | **-0·314, -0·073** | **-0·138*** | **-0·251, -0·024** |
| Constant | -0·061* | -0·108, -0·013 | -0·640*** | -0·740, -0·540 | -1·190*** | -1·304, -1·076 |
| ***Random effects*** |  |  |  |  |  |  |
| Variance (timepoint) | 0·040 | 0·035, 0·045 | 0·040 | 0·035, 0·045 | 0·040 | 0·035, 0·046 |
| Variance (constant) | 0·680 | 0·635, 0·728 | 0·660 | 0·615, 0·708 | 0·458 | 0·418, 0·503 |
| Covariance (timepoint, constant) | -0·113 | -0·127, -0·099 | -0·111 | -0·125, -0·097 | -0·093 | -0·106, -0·079 |
| Variance (residual) | 0·534 | 0·522, 0·546 | 0·534 | 0·522, 0·546 | 0·533 | 0·521, 0·546 |

Verbal and language ability random slopes mixed effects model including an interaction between timepoint and short stature (not significant and not shown), adjusted for sex (Model 1), for sex and birth weight (kg) (Model 2) and for all covariates (Model 3).

Table 16. Multiple imputations: imputed variables and models.

| Variable | Model | Complete | Incomplete | Imputed | Total |
| --- | --- | --- | --- | --- | --- |
| Imputed | | | | | |
| Short stature age 3 | Logistic | 95,725 | 32,333 | 32,333 | 128,058 |
| Ethnicity (White) | Logistic | 127,722 | 336 | 336 | 128,058 |
| Breastfeeding | Logistic | 127,680 | 378 | 378 | 128,058 |
| Second-hand smoke | Logistics | 127,876 | 182 | 182 | 128,058 |
| Mother’s education | Logistic | 127,589 | 469 | 469 | 128,058 |
| Mother’s depression or anxiety diagnosis | Logistic | 127,883 | 175 | 175 | 128,058 |
| Income quintile | Multinomial | 127,484 | 574 | 574 | 128,058 |
| Birth weight | Linear | 127,603 | 455 | 455 | 128,058 |
| Mother’s age | Linear | 127,701 | 357 | 357 | 128,058 |
| Mid-parental height | Linear | 126,763 | 1,295 | 1,295 | 128,058 |
| IMD | Linear | 128,051 | 7 | 7 | 128,058 |
| Weight for age z score | Linear | 90,052 | 38,006 | 38,006 | 128,058 |
| Height for age z score (ages 5 to 11) | Linear | 56,606 | 68,452 | 68,452 | 128,058 |
| Bracken percentile | Linear | 91,658 | 36,400 | 36,400 | 128,058 |
| Age in months | Linear | 94,545 | 33,513 | 33,513 | 128,058 |
| Language score SD | Linear | 53,389 | 74,669 | 74,669 | 128,058 |
| Complete variables | | | | | |
| Sex | - | 128,058 | 0 | 0 | 128,058 |
| Region | - | 128,058 | 0 | 0 | 128,058 |
| Growth-affecting health condition | - | 128,058 | 0 | 0 | 128,058 |

Note: variables highlighted in grey are auxiliary and not included in final models.

Table 17. Multiple imputation estimates: Verbal and language ability random slopes and random short stature mixed effects model including short stature and timepoint interaction effect (n=18,294).

|  |  | F(3, 1232·7) = 21·04  Prob > F < 0·001 |
| --- | --- | --- |
| ***Fixed effects*** | Coef· | 95% CI |
| Timepoint | -0·006 | -0·013, 0·002 |
| Short stature | -0·223*** | -0·328, -0·118 |
| Interaction: short stature # timepoint | -0·001 | -0·036, 0·034 |
| Constant | -0·016 | -0·037, 0·006 |
| ***Random effects*** |  |  |
| Variance (timepoint) | 0·155 | 0·142, 0·170 |
| Variance (constant) | 0·720 | 0·692, 0·748 |
| Covariance (timepoint, constant) | -0·646 | -0·681, -0·609 |
| Variance (residual) | 0·810 | 0·803, 0·818 |

Note: * denotes coefficient is significant at p<0·05 level
** denotes coefficient is significant at p <0·01 level

*** denotes coefficient is significant at p < 0·001 level

| Table 18. Multiple imputation estimates: Verbal and language ability random slopes mixed effects models (n=18,294). | | | | | | |
| --- | --- | --- | --- | --- | --- | --- |
|  | Model 1 | | Model 2 | | Model 3 | |
|  | F(4, 2194·4) = 35·23  Prob > chi2 <0·001 | | F(5, 3140·2) = 77·80  Prob > chi2 <0·001 | | F(17, 15363·0) = 333·29  Prob > chi2 <0·001 | |
| ***Fixed effects*** | Coef· | 95% CI | Coef· | 95% CI | Coef· | 95% CI |
| Timepoint | -0·006 | -0·010, 0·003 | -0·006 | -0·013, 0·002 | -0·006 | -0·013, 0·002 |
| **Short stature** | **-0·225***** | **-0·329, -0·120** | **-0·177**** | **-0·279, -0·075** | **-0·142**** | **-0·238, -0·034** |
| Constant | -0·167*** | -0·204, -0·130 | -0·742 | -0·821, -0·663 | -1·200*** | -1·288, -1·112 |
| ***Random effects*** |  |  |  |  |  |  |
| Variance (timepoint) | 0·155 | 0·142, 0·170 | 0·155 | 0·141, 0·170 | 0·155 | 0·141, 0·170 |
| Variance (constant) | 0·713 | 0·685, 0·741 | 0·701 | 0·673, 0·730 | 0·552 | 0·520, 0·585 |
| Covariance (timepoint, constant) | -0·640 | -0·675, -0·601 | -0·636 | -0·672, -0·596 | -0·650 | -0·694, -0·601 |
| Variance (residual) | 0·810 | 0·803, 0·818 | 0·810 | 0·803, 0·818 | 0·810 | 0·803, 0·818 |

Note: Verbal and language ability random slopes mixed effects model including an interaction between short stature and timepoint (not significant and not shown)· Model 1 is adjusted for sex, Model 2 adjusted for sex and birth weight (kg), and Model 3 for sex, birth weight (kg), ethnicity, mother’s level of education, exposure to second-hand smoke, any breastfeeding, mother’s age at birth of child, mid-parental height SDS, household income quintile, and IMD.

* denotes coefficient is significant at p<0·05 level. ** denotes coefficient is significant at p <0·01 level. *** denotes coefficient is significant at p < 0·001 level.

Table 19. Number analogies activity score at age 17 regression models with standardized beta coefficients (n=7,686).

|  | Model 1· | | Model 2· | | Model 3· | |
| --- | --- | --- | --- | --- | --- | --- |
|  | Coef·  (beta coef·) | 95% CI | Coef·  (beta coef·) | 95% CI | Coef·  (beta coef·) | 95% CI |
| Short stature | -0·747***  (-0·053) | -1·055, -0·438 | -0·594***  (-0·042) | -0·904, -0·284 | -0·418**  (-0·030) | -0·718, -0·118 |
| Constant | 5·759 | 5·673, 5·845 | 4·325 | 3·952, 4·698 | 3·191 | 2·700, 3·681 |

Models are adjusted for sex (Model 1), for sex and birth weight (kg) (Model 2) and for all covariates (Model 3).
